# Structural origins of cartilage shear mechanics

**DOI:** 10.1126/sciadv.abk2805

**Published:** 2022-02-11

**Authors:** Thomas Wyse Jackson, Jonathan Michel, Pancy Lwin, Lisa A. Fortier, Moumita Das, Lawrence J. Bonassar, Itai Cohen

**Affiliations:** 1 Department of Physics, Cornell University, Ithaca, NY, USA.; 2 School of Mathematical Sciences, Rochester Institute of Technology, Rochester, NY, USA.; 3 Department of Clinical Sciences, Cornell University, Ithaca, NY, USA.; 4 Meinig School of Biomedical Engineering, Cornell University, Ithaca, NY, USA.; 5 Sibley School of Mechanical and Aerospace Engineering, Cornell University, Ithaca, NY, USA.

## Abstract

Articular cartilage is a remarkable material able to sustain millions of loading cycles over decades of use outperforming any synthetic substitute. Crucially, how extracellular matrix constituents alter mechanical performance, particularly in shear, remains poorly understood. Here, we present experiments and theory in support of a rigidity percolation framework that quantitatively describes the structural origins of cartilage’s shear properties and how they arise from the mechanical interdependence of the collagen and aggrecan networks making up its extracellular matrix. This framework explains that near the cartilage surface, where the collagen network is sparse and close to the rigidity threshold, slight changes in either collagen or aggrecan concentrations, common in early stages of cartilage disease, create a marked weakening in modulus that can lead to tissue collapse. More broadly, this framework provides a map for understanding how changes in composition throughout the tissue alter its shear properties and ultimate in vivo function.

## Teaser

We present a new framework to understand how changes to the microstructure of cartilage lead to a mechanical phase transition.

## INTRODUCTION

Affecting more than 27 million people in the United States (*1*), and more than 250 million people worldwide, osteoarthritis is one of the leading causes of disability. Osteoarthritis can arise from trauma, mechanical forces, inflammation, biochemical reactions, and metabolic changes to cells (*2*–*4*). As this disease progresses, inflammatory mediators can induce release of enzymes that result in degradation of the extracellular collagen and aggrecan networks (*5*), the two most important constituents responsible for the mechanical properties of cartilage. The networks formed by these constituents are quite distinct in their properties. The collagen molecules form a network of fibrils with very high tensile strength (*6*–*10*). The aggrecan network is composed of highly charged chondroitin sulfate molecules attached to a core protein in a bottle brush geometry. These aggrecan monomers combine with hyaluronic acid to form 10 to 1000 MDa aggregates that gel (*11*). The high amount of charge on the aggrecan network produces an osmotic stress that draws in water and swells the tissue. Broadly, damage to the collagen network leads to loss of tissue integrity (*3*) and a reduction in the capacity of the tissue to resist tensile (*7*) and shear strains (*12*, *13*). Loss of aggrecan reduces the osmotic swelling, makes the tissue more susceptible to compression (*14*), and is also associated with the loss of shear properties (*15*, *16*). As these networks degrade, the tissue mechanics degrade as well (*15*–*17*), though often in a nonintuitive, nonlinear, and depth-dependent manner, until eventually the tissue fails catastrophically. Understanding the path toward mechanical failure in cartilage requires knowledge of how the collagen and aggrecan networks contribute to function under compression and shear in both healthy and damaged tissue.

A major step toward understanding how the collagen and aggrecan networks contribute to the compressive properties of cartilage was the development of poroelastic (*18*) and mixture theories (*19*), which account for water movement through the extracellular matrix, as well as additional theories that describe electrostatic/osmotic contributions to compressive mechanics (*20*, *21*).

These theories have been useful quantitative frameworks (*18*) to understand experimental observations describing how damage to the charged aggrecan network makes the cartilage more susceptible to compression. As the aggrecan network degrades, the osmotic stress driving tissue hydration dissipates and the drainage time scale is substantially shortened because of larger effective pores in the extracellular matrix. This prediction is confirmed by studies that used enzymes such as chondroitinase abc and trypsin to model degradation of the aggrecan network and found that the bulk compressive modulus of a cartilage explant can decrease by up to 50% (*22*) and that the tissue hydraulic permeability can increase up to 15-fold (*14*). Because of their quantitative predictive power, such theories have been important tools for understanding the compressive mechanics of healthy and damaged cartilage.

While these theories are effective at predicting the compressive behavior of the tissue, much less attention has been paid to the shear behavior and its dependence on tissue composition. Pioneering work has demonstrated that electrostatic contributions from the aggrecan network account for a substantial portion of the shear modulus of healthy cartilage because of the rearrangements of the glycosaminoglycan chains that alter the distances between charged groups (*23*). In addition, there are existing models that are able to simulate complex tissue responses to shear mechanical forces (*24*, *25*), though they do not address the microstructural basis for cartilage shear properties. Last, while a recently developed model by our group has shown that the concentration-dependent shear properties of the collagen network are well described by a rigidity percolation model, this model does not address degraded tissue (see Supplementary Materials). Thus, a quantitative framework for the combined contributions of the collagen and aggrecan networks to accurately predict the shear modulus of healthy and degraded cartilage does not exist. Because the shear mechanics of cartilage are of critical importance to its function in joints, developing such a framework to understand the shear mechanics of cartilage and its dependence on both the collagen and aggrecan constituents has been a major goal of the biomechanics community. Here, we propose such a theory that is capable of predicting the local shear modulus accurately on the basis of its composition for accurately recreating the overall mechanical properties of the tissue in the joint.

**Fig. 1.**
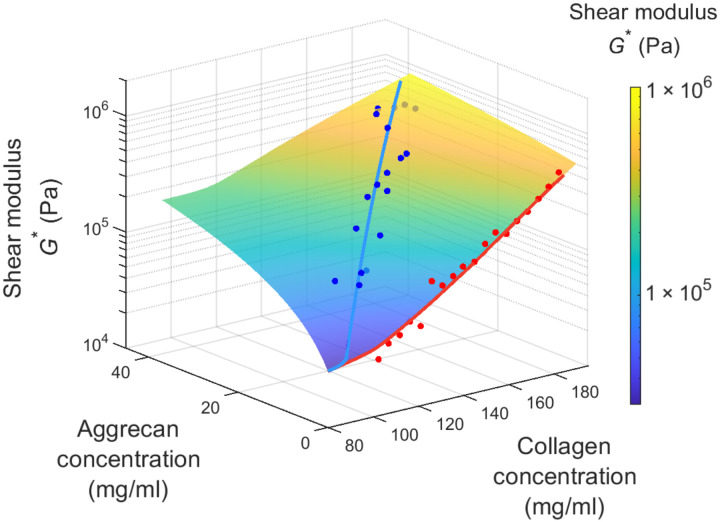
Shear modulus dependence on collagen and aggrecan concentrations: Experiments and rigidity percolation framework prediction. The data points are experimental measurements of the modulus, measured via confocal elastography, as a function of collagen and aggrecan concentrations, measured via Fourier transform infrared imaging (FTIR-I). The surface depicts the theoretical prediction from our rigidity percolation model.

Here, we build on previous work (*13*) and use state-of-the-art experiments and theory to develop a complete rigidity percolation framework for understanding the structural origins of cartilage shear mechanics (Fig. 1). In this instance, rigidity percolation refers to the emergent phase behavior that occurs when a previously sparse network becomes sufficiently connected through additional bonds to propagate shear forces. This approach has been previously used to model cytoskeletal networks yielding important intuition about cell mechanics (*26*, *27*). There are, however, limited examples of its application to describe the mechanics of extracellular matrix in tissues. This framework reveals that even describing the linear shear modulus response of cartilage requires accounting for the critical interplay between the primary collagen and reinforcing aggrecan networks. It predicts that the shear modulus of cartilage is governed by how close the composite network is to the rigidity percolation threshold. Near this threshold, small compositional changes in either aggrecan or collagen drive large changes in shear mechanics, giving rise to a phase transition between a healthy, percolated, and load-bearing network and a degraded, sparse network that can no longer sustain any shear loads. Such predictions yield particularly important insights into the mechanical behavior of the tissue near the cartilage surface where the collagen network is near the rigidity percolation threshold (*13*) and minor changes to the aggrecan network translate into large changes in the shear modulus (*28*).

This paper combines novel experiments measuring local composition and shear mechanics with simulations of rigidity percolation phase transitions to develop and validate this model. Ultimately, this framework will enable predictions of how alterations in tissue structure and composition drive changes in mechanics that occur over the course of disease with the potential to inform diagnosis and therapy.

## RESULTS

### Depth-dependent composition and mechanics

**Fig. 2.**
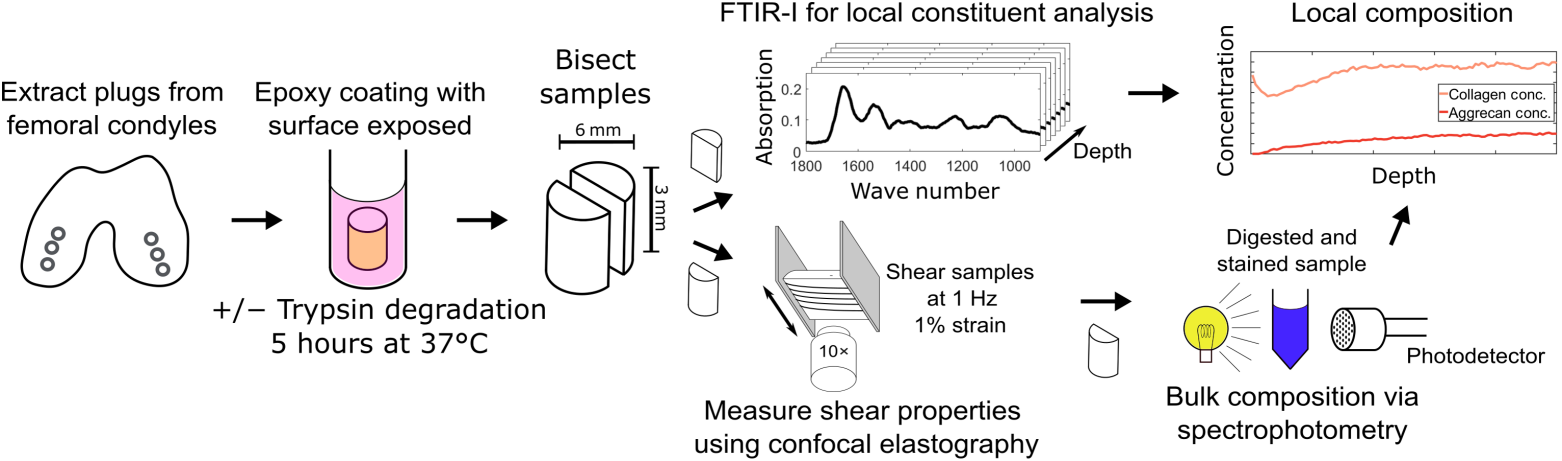
Experimental protocols. Samples are extracted from medial and lateral condyles of neonatal bovid. A subset of the samples are covered in an epoxy coating leaving the surface exposed and submerged in a bath of trypsin for 5 hours at 37°C. This subset constitutes the degraded samples. All samples are then bisected, with half being used for local compositional analysis with FTIR-I, and the other half being used for local mechanical analysis with confocal elastography. After mechanical testing, samples are prepared for biochemical assays used to measure the absolute concentration of the constituents. These measurements are used to calibrate the FTIR data to determine the absolute concentrations of collagen and aggrecan.

The complexity of the behavior predicted by the rigidity percolation model requires a large amount of data relating matched measurements of collagen concentration, aggrecan concentration, and shear modulus. To gather these data, we apply techniques that we developed to measure and register composition and shear properties of cartilage on the microscale (*13*, *29*, *30*). Specifically, we use a combination of Fourier transform infrared imaging (FTIR-I) and confocal elastography on matched samples of bovine cartilage as schematized in Fig. 2. The extracellular matrix of cartilage shows significant variation in collagen concentration primarily near the tissue surface (*13*). By degrading the aggrecan molecules in this region, we can obtain structure-function relationships for the extracellular matrix that span concentrations in both the collagen and aggrecan networks. Toward this end, we harvested cartilage tissue plugs from femoral condyles and subjected a subset of the samples to tissue degradation with trypsin, which degrades the aggrecan core protein so that aggrecan leaves the tissue (*14*). Trypsin is a common enzyme used to assess the effects of aggrecan degradation on cartilage mechanics (*31*–*37*). We then bisected all the tissue plugs. We measured the local tissue composition in one hemicylinder using histology and FTIR-I (*13*). We measured the depth-dependent shear modulus for the matching hemicylinder using confocal elastography (*38*, *39*). Using these measurements on healthy and degraded tissue, we were able to quantify the dependence of the modulus on a wide range of collagen and aggrecan compositions.

### Healthy tissue

**Fig. 3.**
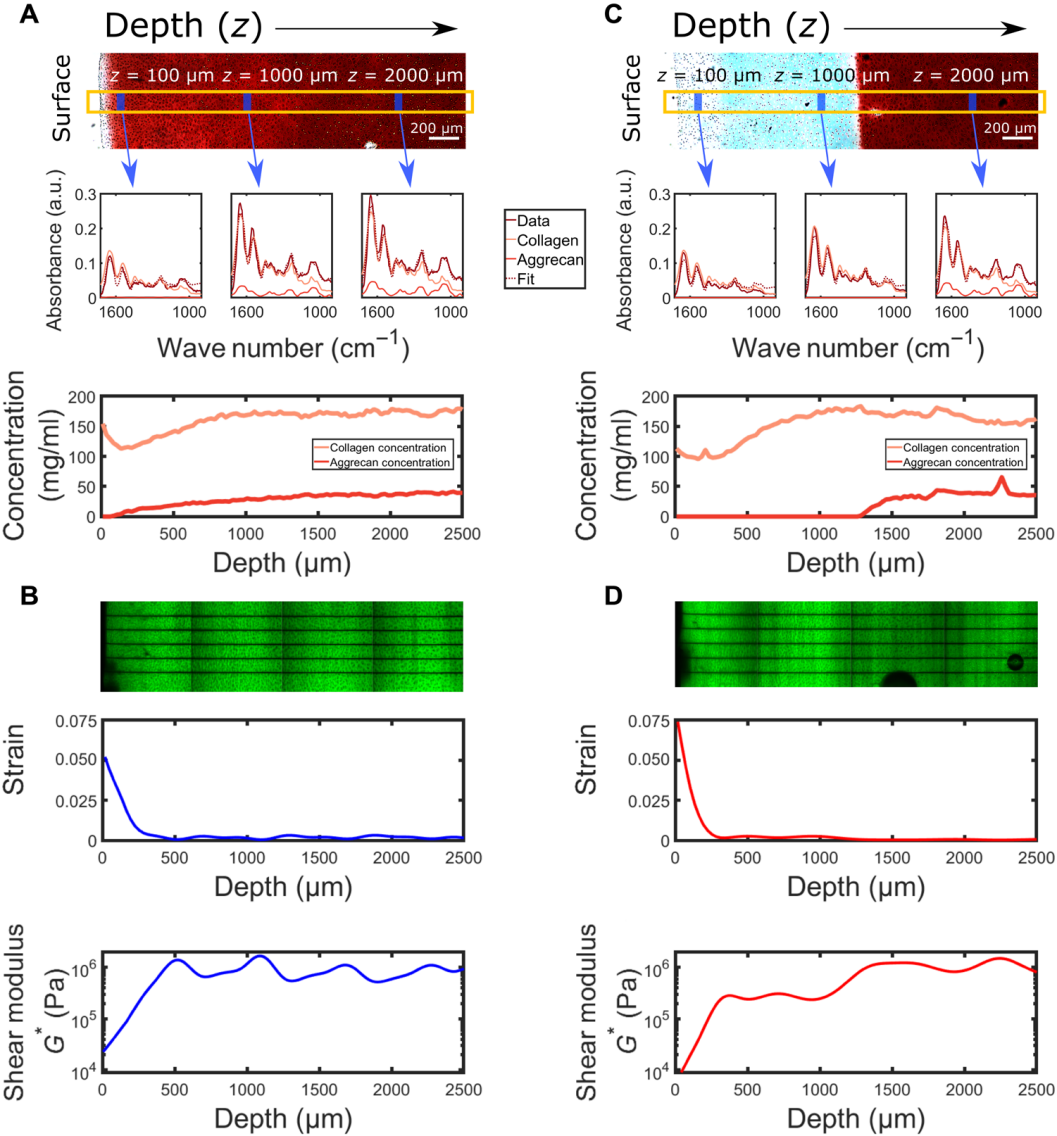
Matched structural and mechanical measurements. (**A**) Composition measurements. Top: Safranin-O–stained histology slides for the healthy and degraded tissue. The areas stained red show regions of proteoglycan content. The yellow box indicates one of three sample spanning sections where the FTIR spectra were taken from, as well as three representative regions at depths *z* = 100 μm, *z* = 1000 μm, and *z* = 2000 μm for which the FTIR spectra are shown. Middle: Measured absorbance spectra along with the fitted aggrecan and collagen contributions. The best-fit spectra are also shown in dashed lines. Bottom: Collagen and aggrecan concentrations versus depth. (**B**) Mechanical measurements. Top: Lines are photobleached perpendicular to the tissue surface and shear oscillations (1% shear strain, 1 Hz) are applied parallel to the surface (Materials and Methods). Middle: By tracking the photobleached lines, we extract the local strain within the tissue. Bottom: These data are combined with measurements of the sample surface area and the total force needed to deform the tissue to extract the shear modulus as a function of depth. The same procedures are repeated for the degraded data to extract (**C**) the collagen and aggrecan concentrations and (**D**) the shear modulus for the degraded tissue as a function of depth.

Results of our depth-dependent histology and FTIR-I measurements for healthy tissue are shown in Fig. 3A. Safranin-O sections showed healthy cartilage morphology with increasing staining with tissue depth suggesting higher aggrecan concentrations deeper in the tissue (Materials and Methods). For each sample, the FTIR spectra as a function of depth were measured as described in Materials and Methods. Sample FTIR spectra at three different tissue depths (*z* = 100 μm, *z* = 1000 μm, and *z* = 2000 μm) are shown in the spectrograms in Fig. 3A. The absorption of IR light as a function of wave number is plotted. The measured spectrum (red) is fit (dashed) by a sum of the contributions from the collagen spectrum (yellow), the aggrecan spectrum (orange), and a linear background (not shown) as described in Materials and Methods. We obtain excellent fits for all the spectra with the aggrecan spectrum contributing most notably to the peak at 1050 cm^−1^. Consistent with the histology results, we find that the aggrecan contribution to the spectra is negligible at the tissue surface and increases with depth.

From the best-fit coefficients and whole-tissue biochemical assays performed on the samples (see the “Biochemical Assay” section for details of the assays), we determined the collagen and aggrecan concentrations with depth (bottom of Fig. 3A). We find that the collagen concentration shows a typical dip just below the cartilage surface (*13*). Specifically, we find that the concentration is 150 mg/ml at the surface, decreases by nearly 30% in the first 100 μm, and then increases again plateauing at 175 mg/ml beyond 800 μm. We find that the aggrecan concentration monotonically increases from having a concentration of 0 mg/ml at the surface to a concentration of nearly 50 mg/ml in the deep zone.

We used confocal elastography to determine the depth-dependent shear modulus of the matching hemicylinder (Fig. 3B). Briefly, we photobleached lines perpendicular to the articular surface and tracked their displacements with depth as described in Materials and Methods. The change in displacement over a given depth is used to determine the depth-dependent strain. Using the extracted strain, surface cross section of the sample, and measurement of the total shear force, we determined the shear modulus G* (Materials and Methods). The measured modulus shows a typical response with a value of about 20 kPa at the surface that increases by almost two orders of magnitude over the first 500 μm and remains roughly constant at greater depths (*38*, *40*, *41*).

### Degraded tissue

Repeating this analysis on degraded tissue (Fig. 3C), we find that the concentration profile of collagen remains unchanged while the aggrecan is completely removed throughout the degraded region. From histology, we observe that aggrecan removal proceeds as a front that is parallel to the tissue surface. The degraded region appears white or light blue while the region of tissue below the degradation front appears deep red. These results are consistent with previous histological measurements of trypsin degraded cartilage samples (*28*).

For each sample, the FTIR spectra as a function of depth were measured as described in Materials and Methods. Sample FTIR spectra at three different tissue depths (*z* = 100 μm, *z* = 1000 μm, and *z* = 2000 μm) are shown in Fig. 3C. Once again, we obtain excellent fits for all the spectra. Consistent with the histology results, we find that the aggrecan contribution to the spectra is negligible in the degraded region as indicated by the absence of the peak at 1050 cm^−1^. From these data and the whole-tissue biochemical assay, we determined the collagen and aggrecan concentrations with depth. We find that the depth-dependent collagen concentration remains similar to that of healthy tissue with a slight dip in concentration just below the surface and higher concentrations in the deeper regions. We find that the aggrecan concentration is close to zero in the degraded region and rises monotonically to levels similar to those in healthy tissues over a 200-μm region at a depth of 1300 μm.

As a result of this degradation, we observe distinct changes to the depth-dependent shear modulus of the matching hemicylinder (Fig. 3D). We observe that the shear modulus at the surface is lower than that in the healthy sample. For depths 400 μm < *z* < 1000 μm, we observe an intermediate value of the modulus that is about an order of magnitude larger than that at the surface and an order of magnitude lower than the modulus in the deep zone. The modulus in the deep zone is very similar to that of healthy tissue, because the degradation front never reaches this region.

### Average depth-dependent composition and mechanics

**Fig. 4.**
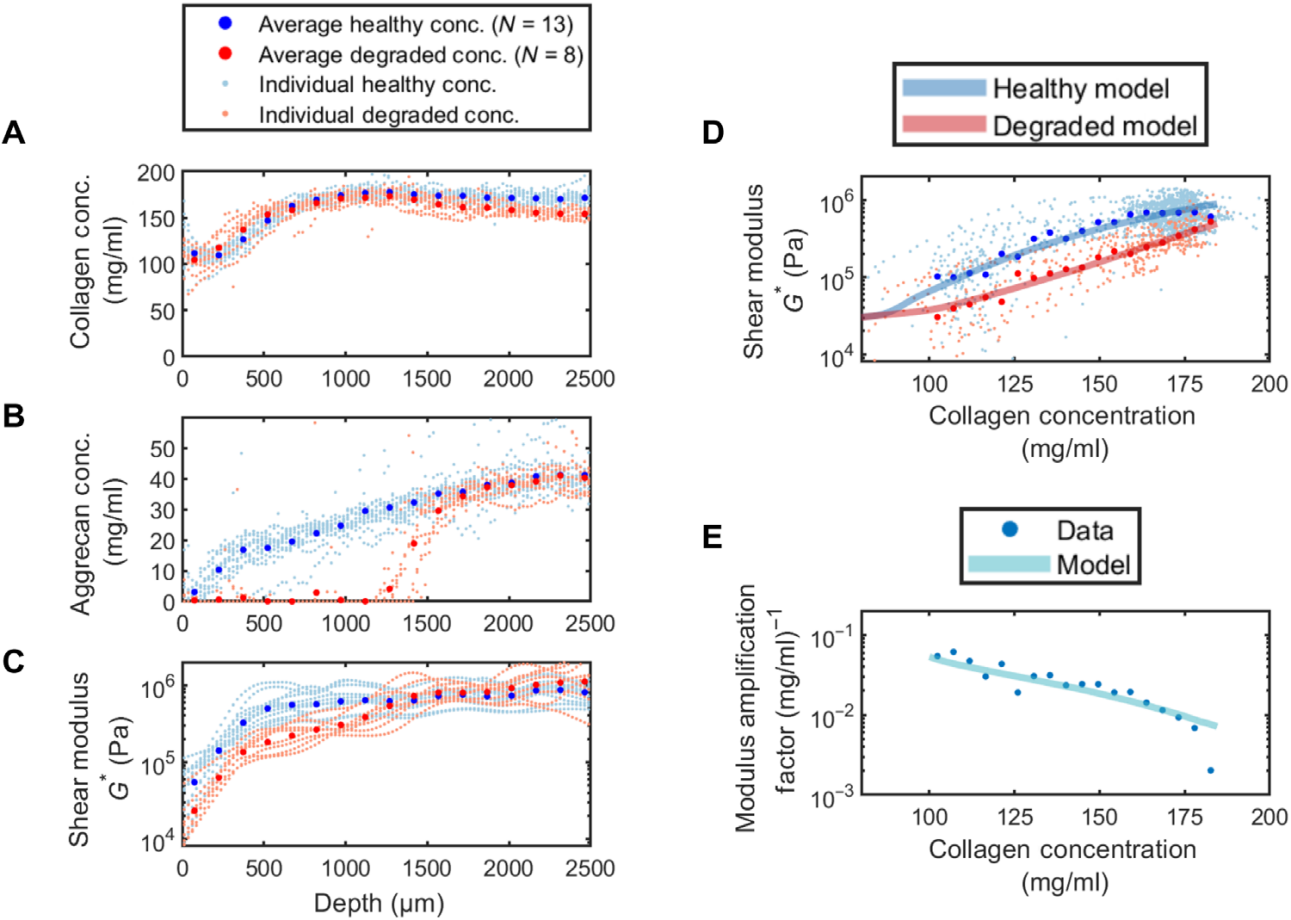
Depth-dependent results and shear modulus dependence on collagen concentration. (**A**) Collagen concentration with depth. Near the tissue surface, there is significant variation in collagen concentration, until it plateaus after a depth of 1000 μm. There is no significant difference between the healthy and degraded samples. (**B**) Aggrecan concentration with depth. In the healthy samples, there is a natural increase in aggrecan concentration throughout the depth of the tissue. In the degraded samples, the aggrecan concentration is zero until the depth of the degradation front, where it returns to a similar concentration as the healthy samples. (**C**) Shear modulus with depth. The shear modulus at the surface of the degraded tissue is lower than in the healthy tissue. In the region 400 μm <*z*< 1000 μm, there is an intermediate value of the modulus that is about an order of magnitude larger than that at the surface and half an order of magnitude lower than the modulus in the deep zone. The modulus in the deep zone is very similar to that of healthy tissue, because the degradation front never reaches this region. (**D**) Shear modulus as a function of collagen concentration. The depth-dependent measurements of the shear modulus, collagen concentration, and aggrecan concentration are combined to create a plot of shear modulus versus collagen concentration for both the healthy and the degraded tissue. The degraded tissue modulus is lower by close to a factor of 5. (**E**) The modulus amplification factor as a function of collagen concentration defined in Eq. 1. The contribution of the aggrecan to the shear modulus is highest at low collagen concentrations and rapidly decreases with increasing collagen concentration.

These trends in the tissue composition and mechanics for the healthy and degraded tissues hold when averaged across multiple samples (*N* = 13 healthy and *N* = 8 degraded). We plot the average collagen composition versus depth for both healthy and degraded samples in Fig. 4A. We find no statistical difference between the healthy and degraded conditions. We plot the average aggrecan concentration versus depth for both healthy and degraded samples in Fig. 4B. As in the single sample data in Fig. 3, we find that aggrecan is completely removed from the fully degraded region for depths *z* < 1200 μm, indicating that our degradation protocol (exposure to trypsin at 0.25% for 5 hours at 37°C) produces consistent results. Following this fully degraded region, we observe a transition region where the aggrecan rapidly increases in concentration until it reaches that of the healthy tissue and the two samples are statistically indistinguishable. We plot the shear moduli for the healthy and degraded samples in Fig. 4C. We observe a consistent downward shift of roughly half an order of magnitude between the averages of the healthy and degraded samples. Moreover, the averages for the degraded samples continue to show an intermediate plateau region with a value between those of the surface and deep zones. While the modulus reduction is roughly constant across the degraded region, the difference in aggrecan concentration between the healthy and degraded tissues varies substantially. These data indicate that the dependence of the modulus on aggrecan varies with depth and depends on collagen concentration.

### Structure-function relationships

To determine the dependence of the modulus on both the aggrecan and collagen concentrations, we plot the local shear modulus as a function of collagen concentration for both the healthy (blue) and aggrecan degraded (red) tissues in Fig. 4D. For the degraded tissue, we only include data with aggrecan concentration below 2 mg/ml since, above this value, the tissue has not been degraded and has the properties of healthy tissue. We find a uniform factor of 5 decrease in the complex modulus for the degraded tissue, which primarily arises from the change in the storage modulus (see the Supplementary Materials for details of loss modulus).

To ascertain the sensitivity of the modulus to aggrecan, we must account for the fact that the aggrecan concentration varies by almost an order of magnitude across these datasets. For example, at high collagen concentrations associated with the deep zone, reducing the aggrecan concentration from 30 mg/ml to zero reduces the modulus by a factor of 5. This factor of 5 reduction in modulus results from reducing the aggrecan concentration from 3 mg/ml to zero near the surface region. To understand this dependence, we define the aggrecan amplification factor (*A*_Agg_)

$$A_{\text{Agg}}(\text{Collagen Conc}.)=\frac{{\text{log}}_{10}(G_{\text{Hel}}^{*})-{\text{log}}_{10}(G_{\text{Deg}}^{*})}{\Delta_{\text{Agg Conc}.}}$$ (1)

where $G_{\text{Hel}}^{*}$, $G_{\text{Deg}}^{*}$ are the shear modulus of the healthy and degraded tissue, and Δ_Agg Conc._ is the difference between the aggrecan concentrations of the measurements for the same collagen concentrations. When the amplification factor (*A*_Agg_) is high, aggrecan plays a more important role in contributing to the shear modulus of the tissue. Conversely, when the amplification factor is low, the aggrecan concentration has a diminished role in determining the modulus.

We plot the modulus amplification factor as a function of collagen concentrations in Fig. 4E. We find that the contribution of aggrecan to the shear modulus is highest at low collagen concentrations and decreases by over an order of magnitude over a less than twofold change in collagen concentration. These measurements are consistent with a rigidity percolation framework to explain the shear mechanics of cartilage. At low collagen concentrations, the network is less likely to percolate on its own and addition of aggrecan increases the likelihood of percolation and hence markedly increases the shear modulus. At higher concentrations of collagen where the network is more likely to be percolated, the presence of aggrecan makes a much smaller contribution to the shear modulus of the tissue.

### Rigidity percolation model

**Fig. 5.**
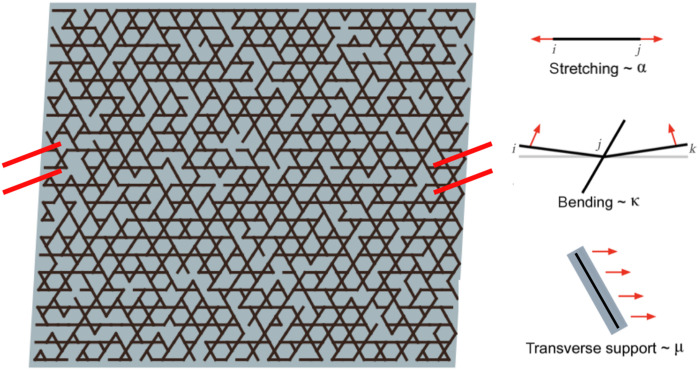
Rigidity percolation model. Shown is a schematic of a portion of the simulated kagome network. Hash marks indicate continuous boundary conditions in the lateral dimension. The black links represent the collagen fibers and the gray represents the background aggrecan gel. The links or bonds in the network are characterized by a stretching modulus α and a bending stiffness κ. The background gel further couples the links in the network through inhibition of transverse transport. This gel is characterized by a modulus μ.

To quantitatively assess the degree to which our framework can describe both the healthy tissue and aggrecan-depleted tissue moduli, we fit the data in Fig. 4 (D and E) to a rigidity percolation model. The model consists of a disordered kagome lattice representing the stiff primary collagen network embedded in a continuum elastic background gel representing the reinforcing aggrecan and hyaluronic acid networks (Fig. 5). The fibers in the collagen network are randomly removed with a probability 1 − *p*, where 0 < *p* < 1, leaving a remaining network of occupation fraction *p*. Each bond is characterized by a stretching modulus α and a bending modulus κ. In a manner similar to (*13*), we include a background gel of aggrecan and other matrix components that resists bond deformations in the transverse direction and has an elastic modulus μ. These additional mechanical constraints lead the network to undergo rigidity percolation at *p_c_* ∼ 0.5 (*13*). In this model, to account for the fact that trypsin primarily affects mechanics through degradation of the aggrecan, we take μ to linearly depend on the concentrations of aggrecan, hyaluronic acid, and collagen (see Materials and Methods). The energy cost of deforming this composite network in the linear response regime is given by

$$\begin{array}{cc} E= & \frac{\alpha }{2}\sum_{\langle \mathrm{ij}\rangle }p_{\mathrm{ij}}{\left(u_{\mathrm{ij}}\cdot {\hat{r}}_{\mathrm{ij}}\right)}^{2}\\ & +\frac{\kappa }{2}\sum_{\langle \mathrm{ijk}\rangle }p_{\mathrm{ij}}p_{\mathrm{jk}}{\left[(u_{\mathrm{ji}}+u_{\mathrm{jk}})\times {\hat{r}}_{\mathrm{ji}}\right]}^{2}\\ & +\frac{\mu }{2}\sum_{\langle \mathrm{ij}\rangle }p_{\mathrm{ij}}[u_{\mathrm{ij}}^{2}-{\left(u_{\mathrm{ij}}\cdot {\hat{r}}_{\mathrm{ij}}\right)}^{2}]+\frac{\mu }{2}\epsilon_{s}^{2}A, \end{array}$$ (2)

where the terms correspond to the energy penalty for fiber stretching, fiber bending, the coupling of the network to the background gel, and the deformation of the background gel, respectively. In the final term, ϵ*_s_* denotes an affine background shear strain, while *A* is the area of the network. The indices *i*, *j*, *k* refer to sites (nodes) in the lattice-based network, such that *p_ij_* is 1 or 0 when a bond between those lattice sites is or is not present. The quantities ${\hat{r}}_{\mathrm{ij}}$ and **u***_ij_* = **u***_i_* − **u***_j_* are, respectively, the unit vector along a bond *ij* and the corresponding relative displacement.

To determine the network properties, we strain the network, use QR decomposition of a constrained stiffness matrix derived from the deformation energy (Eq. 3) to mechanically equilibrate the network, and extract the shear modulus (see Materials and Methods). Specifically, for each set of parameters (α,κ,μ), a network containing ∼10^5^ nodes was randomly generated with a fraction 1 − *p* of bonds missing, subjected to a compressive strain of 5% and a shear strain of 1% applied via the top boundary, and then allowed to relax via fiber deformations, with periodic boundary conditions imposed along the left and right sides of the network. With these simulations, we obtained the modulus *G*_n_ as a function of the bond occupation probability *p* and the gel modulus μ.

To map the results of the simulation to experiments, we linearly scale the network modulus *G*_n_, bond occupation probability *p*, and gel modulus μ to the experimentally measured modulus, collagen concentration, and aggrecan concentration, respectively. The linear mappings for gel shear modulus and bond occupation probability amount to taking the leading order term in a Taylor series, and we truncate at this order as we are already able to achieve strong quantitative agreement. We fit the model to the collagen-dependent modulus and amplification factor data in Fig. 4 (D and E) and find excellent agreement. In all, the rigidity percolation predictions for both the healthy and degraded datasets are obtained using five fitting parameters. Moreover, the fits for the bending-to-stretching ratio κ/α and the modulus of the bare hyaluronic acid gel are in very close agreement with literature values (*23*, *42*–*44*). Once these parameters are fit to the data, it is possible to use this rigidity percolation framework to quantitatively predict the modulus for arbitrary values of the collagen and aggrecan concentrations as shown in Fig. 1.

## DISCUSSION

By collecting a large amount of matched experimental data for local composition and local shear mechanics and fitting them using simulations, we have constructed a rigidity percolation framework that provides valuable insights into the dependence of cartilage mechanical properties on the tissue constituents (Fig. 1). Our measurements and simulations show that the contribution of aggrecan to the shear modulus is highly dependent on the concentration of collagen. When the collagen concentration is high, the aggrecan provides a relatively minuscule contribution to the modulus. Conversely, aggrecan plays a critical role in enhancing the shear properties of cartilage in regions of the extracellular matrix where collagen concentration is low (Fig. 4E). By capturing the interactions between the supporting aggrecan gel and the collagen network, the rigidity percolation framework provides important intuition for the origins of this nonlinear and unusual behavior. When the collagen network is sufficiently concentrated that it percolates by itself, the relative contribution of aggrecan is small. Conversely, when the collagen network is close to the rigidity percolation threshold, even a small reinforcement of the network by aggrecan makes it easier for the composite network to transmit stresses. In combination, the model and experimental data provide a powerful framework for understanding how the shear mechanics of cartilage arise from the interactions of the collagen and aggrecan networks.

The rigidity percolation framework for describing how collagen and aggrecan interact to determine the linear shear mechanics of articular cartilage is remarkably effective despite its simplicity. The model uses a conventional two-dimensional (2D) kagome lattice structure to represent the 3D cross-linked collagen network that is coupled to a background reinforcing network representing the contributions of aggrecan and other matrix components. The contributions of the collagen network concentration, connectivity, and cross-linking are effectively described by the bond occupation probability, *p*, and the lattice structure. The applicability of the 2D kagome network is consistent with its ability to describe other 3D networks of collagen (*13*) and other cytoskeletal networks (*45*) where the number of filaments crossing at the nodes is approximately 2.

A critical contribution of the reinforcing background gel is to provide a coupling between the fibers in the network that lowers the rigidity percolation threshold, *p_c_*. As such, its contribution is not only additive as has been suggested in prior literature (*23*). As shown in the Supplementary Materials, when we exclude the third term in Eq. 3, which couples the network deformations to the background gel, we obtain inferior fits and unreasonable values for the fitting parameters. These include a bending-to-stretching ratio that approaches 1 and a value for the hyaluronic acid gel contribution to the modulus ∼1 Pa, which is three orders of magnitude too small (*11*). These findings highlight that the additional constraints introduced by the aggrecan gel can markedly alter the shear properties of cartilage by helping to drive the composite network through its rigidity percolation transition. This coupling between the two networks may result from stretch-stiffening of the collagen network because of swelling induced by the aggrecan molecules, causing previously buckled collagen fibers to become engaged and contribute mechanically (*21*). Alternatively, the coupling could simply be the aggrecan matrix filling space and reducing the free movement of the collagen fibrils. Further studies are necessary to identify the exact origin of this coupling. This mechanism for controlling the tissue properties is important biologically since the turnover rate for aggrecan is orders of magnitude faster than the turnover rate for collagen. As such, fabrication and degradation of aggrecan by chondrocytes, the cells in cartilage, can be used to rapidly alter the tissue shear mechanics in response to changes in shear loads.

In the present study, we chose trypsin treatment to interrogate the role of the reinforcing background gel. Although the primary effects of trypsin treatment is proteoglycan removal, it is possible that changes to other extracellular matrix components occur. To assess whether such changes affected the mechanics of the primary network, we performed additional model fits directly on the degraded samples and compared them to fits performed on the entire dataset. We found no differences in the collagen-related parameters α and κ. As such, we are confident that, in this system, trypsin treatment did not alter the collagen network sufficiently to change its mechanics. Future studies using different enzymes that target other structural aspects of either the collagen or aggrecan networks would be helpful in elucidating the relationship between the various parameters in our model and components of the extracellular matrix.

A model that describes the dependence of cartilage shear mechanics on composition is a powerful tool for understanding the progression of diseases of cartilage such as arthritis. Such diseases develop over many years and are typically characterized by slow degradation of one or more components of the extracellular matrix, but at late stages often result in rapid loss of joint function due to compromised cartilage mechanics. In the context of the proposed framework, this rapid loss of function may arise from the rapid decrease in mechanical integrity associated with crossing the rigidity percolation threshold. Recent advances in clinical magnetic resonance imaging of cartilage tissue enable mapping of collagen and aggrecan composition and organization in vivo (*46*). Relating such measurements to tissue and joint scale mechanics would be a major advance in the field. While the present work was performed on neonatal bovine cartilage, the orders of magnitude change in shear modulus in depth is also seen in adult equine (*47*), adult human ankle (*48*), and adult human knee cartilage (*39*, *48*). Thus, it is likely that the core predictions of the model are more broadly applicable. As such, the rigidity percolation framework presented here may allow for identifying tipping points during disease progression where small additional changes in composition lead to tissue failure. This understanding could be critical for informing treatment by identifying stages of disease progression that are most in danger of compromising function due to loss of collagen, aggrecan, or both.

In addition, this framework provides new insights for understanding and designing cartilage therapies. Specifically, regenerative medicine approaches frequently involve delivery of cells alone (*49*) or in combination with weak scaffolds (*47*, *50*) to promote cartilage regeneration. Critical to the success of such approaches is the generation of a new mechanically competent extracellular matrix. Cells alone or those embedded in sparse matrices are likely far below the rigidity percolation threshold and, as such, will take a substantial amount of time to achieve mechanical competence. The framework described here suggests that designing implants close to the rigidity percolation threshold will maximize the impact of cellular matrix biosynthesis on their mechanical performance.

A critical parameter for developing such scaffolds is the thickness of the fibers. Thinner fibers will generate more bonds per unit mass and be more likely to form a percolated network than thicker fibers for the same total concentration of collagen (*51*). An excellent example is reconstituted collagen gels, which have relatively small fiber diameters (∼50 nm) (*52*) and have a modulus at relatively low mass concentrations (3 mg/ml). In contrast, since collagen fibers in cartilage are thicker (∼1 μm), the concentration of collagen required for rigidity percolation in tissue extracellular matrix is significantly larger (∼90 mg/ml) than that in reconstituted collagen gels. Within our model, such differences are captured by the bond occupation probability *p* and its relationship to collagen concentration δ.

While the model presented here is purposefully simple to emphasize the origins of the mechanical phase transition seen in cartilage, it could easily be modified to address additional important properties of cartilage. For example, cartilage is known to exhibit important rate dependence in shear (i.e., viscoelasticity). To address viscoelasticity, the model could be extended by adding a rate-dependent stress in the background gel or in the bond elements. A sufficiently viscous response would enhance coupling between bond elements and could result in a frequency-dependent rigidity percolation threshold. In addition, while collagen orientation is not correlated with the tissue modulus in the linear regime (*13*), such network properties are expected to contribute to the tissue response in the high strain limit. It is therefore likely that the model will need to be modified to account for additional extracellular matrix properties to address the high strain regime where phenomena such as strain stiffening are important. Last, while previous work has demonstrated that 2D and 3D filamentous lattice models exhibit very similar scaling of their elastic moduli with increasing connectivity, it would nevertheless be interesting to determine whether extensions to 3D networks give additional insight into the observed mechanical phase transition. Overall, implementing such modifications to the model would enable broader applicability and should also enable probing of how more complex loading modalities that include compression and extension alter the mechanical phase transition we have reported here.

Overall, we developed a rigidity percolation framework to understand the structural origins of cartilage shear mechanics on the basis of the composition and interactions of the collagen network and the reinforcing aggrecan gel that together account for most of the tissue extracellular matrix properties. Since these are also the main constituents of all extracellular matrices in connective tissue in mammals, this framework and its extensions into nonlinear deformation regimes may be a widely applicable tool for understanding the mechanics of many if not all connective tissues in health, disease, and repair (*50*). Similarly, this framework may also prove useful for understanding artificial constructs with tissue-like properties (*50*). More broadly, this work illustrates a notable example of how biology exploits compositional perturbations driving physical processes at the proximity of phase transitions to achieve remarkable function in tissue homeostasis, disease, and repair.

## MATERIALS AND METHODS

### Tissue harvesting

One- to three-day-old neonatal bovine knee joints (*N* = 11) were acquired from a local abattoir (Gold Medal Packing, Rome, NY). Neonatal cartilage was chosen because of its similarity in shear modulus profile to human cartilage (*39*, *53*), as well as ease of access, consistency of tissue samples, and prior use in similar studies (*13*, *28*, *38*–*41*). Cylindrical explants 3 mm in height and 6 mm in diameter were dissected from the medial and lateral femoral condyles.

### Enzymatic degradation

Samples were enzymatically degraded by immersing them in a bath of trypsin-EDTA 0.25% (Sigma-Aldrich) at 37°C for 5 hours (Fig. 2). Epoxy glue was used to create a protective coating around the sample edges, with the top surface of the tissue left exposed. This procedure allowed for creating a well-controlled degradation front. Trypsin was chosen for its ability to primarily affect mechanics through degradation of the aggrecan and minimal effect on the collagen network (*34*). After degradation, the samples were rinsed with phosphate-buffered saline (PBS), the glue was peeled off, and the samples were bisected into two hemicylinders. One hemicylinder was placed in protease inhibitors for mechanical testing, and the other was fixed in 10% PBS-buffered formalin for compositional analysis via FTIR-I.

### Histology

Qualitative analysis of aggrecan removal was conducted via Safranin-O staining as described previously (*28*). Tissue samples were fixed in neutral buffered formalin, embedded in paraffin, cut into 4-μm-thick sections, and placed onto glass slides. Sections were dewaxed in three xylene baths for 2 min each and rehydrated in three baths of ethyl alcohol (100, 95, and 70% ethanol, appropriately diluted with distilled water) for 2 min each. The nuclei were stained with Weigert’s iron hematoxylin for 10 min and then the samples were rinsed for 10 min in running water. The samples were then stained with fast green solution for 5 min and rinsed with 1% acetic acid solution for 15 s. Last, the samples were stained with 0.1% Safranin-O (pH 2.0) solution for 8 min.

### Compositional measurements

Quantitative measurements of collagen and aggrecan relative concentrations were obtained via FTIR-I similarly to previous studies (*13*, *29*, *30*, *54*, *55*). Sections, 4-μm thick from each tissue sample, were placed on 2-mm-thick mid-IR transparent BaF_2_ disks that were 25 mm in diameter (Spectral Systems, Hopewell Junction, NY). Although the sectioning plane was fixed, the section cutting direction was randomized to prevent systematic biases in section thickness due to cutting. Sections were dewaxed in three xylene baths for 2 min each and rehydrated in three baths of ethyl alcohol (100, 95, and 70% ethanol, appropriately diluted with distilled water) for 2 min each.

Samples were loaded into a Hyperion 2000 FTIR-I microscope (Bruker, Billerica, MA) in transmission mode and we acquired data for wave numbers between 600 and 4000 cm^−1^ with a resolution of 4 cm^−1^. A 15× objective was used with a slit aperture configured to acquire spectra over a rectangular region 25 μm by 200 μm, where the long dimension was parallel to the articular surface. Fifteen background-corrected scans were repeated at a given measurement point and averaged to generate a single IR spectrum. The acquisition window was scanned along the tissue sample’s depth at 25-μm intervals by a computer-controlled stage to acquire measurements throughout the depth of the tissue. This procedure was repeated three times for each tissue sample with each scan separated laterally by roughly 2 mm taking care to avoid blood vessels and other artifacts.

We obtained the relative concentrations of aggrecan and collagen from the measured spectra by fitting the spectral window between 900 and 1725 cm^−1^ (*13*, *30*) for each measurement point with a linear combination of previously measured spectra for collagen and aggrecan (*55*), as well as a linear background. This method is based on Beer-Lambert’s law, which states that IR absorbance is proportional to molecular concentration, and two mixed species of molecules have additive contributions. Including a linear background correction accounts for the instrument-specific deviations and drift, which can occur in different laboratories for different environmental conditions (*56*). In fitting the spectra, we constrained the relative concentration of aggrecan and collagen to be greater than zero and assume that the linear background is the same throughout each sample. We did not consider the trace amounts of type IX collagen, type XI collagen, elastin, small nonaggregating proteoglycans, and other matrix macromolecules that occur in cartilage as their limited contributions to the absorption spectrum were negligible (*57*). The aggrecan and collagen concentration coefficients of the scans were then averaged for each depth, to determine the relative collagen and aggrecan concentrations as a function of depth.

### Mechanical measurements

To prepare for mechanical testing, each hemicylindrical sample was soaked for 1 hour in PBS with 5-DTAF (5-dichlorotriazinylaminofluorescein; 7 mg/ml; Life Technologies, Carlsbad, CA), an all-protein stain. Samples were rinsed in PBS for 30 min to remove excess dye and then loaded into a Tissue Deformation Imaging Stage (Harrick Scientific, Pleasantville, NY). In this apparatus, the sample is gripped between two plates. Shear oscillations are applied to the surface of the sample, and the displacement of the second plate is used to measure shear stresses (*39*, *40*). The sample was held in place between the shearing plates using cyanoacrylate glue to eliminate the need for compression. Over the course of the experiments, average compressive strain was found to be less than 0.03%. Samples were immersed in PBS to maintain tissue hydration during mechanical testing.

The Tissue Deformation Imaging Stage (Harrick Scientific) was mounted onto an inverted LSM 5 Live confocal microscope (Carl Zeiss, Jena, Germany), where five lines were photobleached onto the rectangular surface of the hemicylinder perpendicular to the tissue surface. These lines caused no damage to the tissue and were used to facilitate automated computer tracking of the strain with a depthwise resolution of 10.4 μm. Imaging of 1 Hz oscillatory shear at 1% peak strain amplitude was carried out with a 10× objective, and movies of the tissue oscillations were acquired at 20 FPS throughout the entire depth of the tissue (Fig. 2). All mechanical testing was performed within 48 hours of tissue harvest. The samples were retrieved after mechanical testing and frozen for biochemical assay.

To isolate the effects of aggrecan on the tissue properties, our protocol entailed conducting our shear experiments under no compression. This procedure was necessary to avoid any effects because of buckling of the collagen network (*38*), which would be much more localized in the degraded tissue. The main consequence of this procedure was that we observed much smaller changes in the moduli and energy dissipation for the degraded tissue than those observed in (*13*, *28*). Avoiding this complication was also important for testing the rigidity percolation model since the model does not yet account for buckling of collagen fibers under compression. Since the normal function of cartilage tissue does entail compression, extending the model to address this regime would also be important.

Automated tracking of the local shear strain was facilitated by the five photobleached lines along the depth of the tissue. A custom Matlab code tracked the amplitude of the oscillations of the tissue, and the local shear strain was calculated by numerically differentiating the displacement with respect to the depth (*40*). The strain curves were fit to a sinusoidal function. The force applied to the tissue was able to be calculated by observing the displacement of the stationary shearing plate of a known stiffness. The depth-independent shear stress was able to be calculated by dividing this force by the surface cross section of the tissue. The shear modulus is this shear stress divided by the local shear strain. The difference in phase of oscillations between the local region and the applied stress is also recorded, which provides a measure of the viscous shear modulus relative to the total shear modulus.

### Biochemical assay

To determine the absolute concentrations of collagen and aggrecan in a tissue sample, we calibrated the coefficients obtained from the FTIR-I measurements using biochemical analysis (*58*). Mechanically tested hemicylinders were weighed for their wet weight, and frozen at −80°C. The samples were dehydrated in a lyophilizer for 48 hours and then weighed again to obtain the dry weight. The samples were incubated in papain digest buffer (PDB) made from papain (125 mg/ml; Sigma-Aldrich) and 10 mM *N*-acetyl cysteine (Sigma-Aldrich) in PDB buffer (100 mM phosphate and 10 mM EDTA, pH 6.5) for 12 hours at 60°C.

To measure the aggrecan concentration, GAG standards were created by dissolving chondroitin sulfate in PDB to known concentrations from 2 to 250 μg/ml. Fifty microliters of each standard and 50 μl of the digest solution were added to a 96-well plate. Two hundred fifty microliters of pH 3 DMMB dye (Sigma-Aldrich) was added to each well. The plate was shaken for 30 s and then the absorbance was read at 525 nm. The absorbance of the samples was matched to the absorbance of the known standards.

To measure the collagen concentration, hydroxyproline standards were created by dissolving hydroxyproline in PBD to known concentrations from 2 to 1000 μg/ml. Fifty microliters of 2 M NaOH was added to 50 μl of each standard and sample. The standards and samples were heated to 110°C for 18 hours. A total of 30.5 μl of HCl solution, 100 μl of 0.001 M CuSO_4_, 100 μl of 2.5 M NaOH, and 100 μl of 6% H_2_O_2_ were each added to the standards and samples, ensuring to vortex after each addition. The standards and samples were allowed to rest at room temperature for 2 hours. The samples were vortexed and heated to 80°C for 5 min and then frozen. Four hundred microliters of 3 M H_2_SO_4_ was added to each standard and sample and then they were frozen again. Two hundred microliters of DMAB (Sigma-Aldrich) was added to each standard and sample and then they were heated at 70°C for 15 min. Two hundred microliters of each standard and sample was added to a 96-well plate, and the absorbance was read at 540 nm. The absorbance of the sample was matched to the absorbance of the known standards. Collagen is approximately 13.5% hydroxyproline, so the measure of hydroxyproline was multiplied by 7.4 to find the concentration of collagen in the sample.

The total concentrations of collagen and chondroitin sulfate were taken from the biochemical assays and divided by the volumes of each sample, approximated by the depth of the sample measured during mechanical testing multiplied by its surface cross section. The concentrations were averaged for the healthy samples, and the degraded samples and the FTIR-I curves were calibrated to the total average concentrations (*30*).

### Mathematical model

Rigidity percolation theories model biopolymer networks as disordered fiber networks and provide a framework to connect their rigidity to the network structure, composition, and single-filament properties (*59*–*64*). These models have been immensely successful in predicting the mechanical properties and phase transitions of in vitro cytoskeletal and extracellular matrix networks as a function of filament concentrations. Previously, we combined this framework with a lattice-based disordered fiber network reinforced by a background gel to explain the depth-dependent shear properties of AC (*13*). In this framework, the shear modulus of the tissue was taken to be a sum of a background shear modulus, μ, and a simulated shear modulus, *G_sim_*(κ/α, μ/α, *p*), where κ and α denote the bending and the stretching moduli of the collagen fibers, and *p* is the bond occupation probability of the collagen network, respectively. While this model simplifies the collagen network by assuming uniform stiffness and thickness of the fibrils, which fall within literature values (*43*), the exact values of κ and α do not affect the critical bond fraction or fiber density at which the rigidity percolation phase transition takes place. Here, we adapted this model to predict the mechanical response of both healthy and enzyme degraded articular cartilage.

Since degradation with trypsin removes the aggrecan leaving the background hyaluronic acid network nearly intact, we extended the model to describe separate contributions of aggrecan and the associated hyaluronic acid network. Furthermore, the background modulus also includes mechanical reinforcement from collagen fibers that do not participate in the formation of a percolated network. Therefore, we expand μ about the bare HA value μ_0_, to linear order in aggrecan and collagen concentrations, ρ*_a_* and ρ*_c_*, respectively, as μ = μ_0_ + βρ*_a_* + γρ*_c_*, where β and γ are expansion coefficients capturing the reinforcement of the bare hyaluronic acid network by aggrecan and collagen. We computed the full shear modulus as

$$G=c[\mu +G_{\text{network}}(\kappa /\alpha ,\mu /\alpha ,p)]$$ (3)

$$=c[\mu_{0}+\beta \rho_{a}+\gamma \rho_{c}+G_{\text{network}}(\kappa /\alpha ,\mu /\alpha ,\delta \rho_{c})]$$ (4)

where *c* is a scaling factor from simulation to experimental units, and the network modulus *G*_sim_ was obtained from the simulations by minimizing the deformation energy density of the system, E, under a given small strain ϵ*_s_* and calculating $\frac{\partial^{2}E}{\partial {\epsilon_{s}}^{2}}$ (see the Supplementary Materials for model construction and simulation details).

The lattice-based network is diluted sufficiently by removal of bonds such that the resulting disordered network has a broad distribution of fiber lengths as in disordered networks in real tissues. The kagome lattice was selected because for a network based on such a lattice, the maximum coordination number is 4, i.e., one will never have more than two crossing fibers at any cross-link; this is also true for disordered extracellular matrix networks in tissues. At the same time, since the fibers are stiff (i.e., both fiber stretching and fiber bending cost energy), such a network can also have a finite shear modulus (above the rigidity percolation threshold) despite having a maximum coordination number of 4. The random, uncorrelated bond dilution does create a network that is isotropic and does not account for tissue depth-dependent fiber alignment seen in articular cartilage (*65*). While such anisotropies may play an important role in tissue response to large externally applied shear strains, previous work from our group has shown that fiber alignment does not play a critical role in determining tissue shear mechanical properties in the linear response regime (*13*).

### Determination of model parameters

We assumed the collagen fibers to be rods with a cross-sectional radius *r* ∼ 10 nm (*42*) and Young’s modulus *E* ∼ 1 GPa (*43*). In the simulations, lengths and displacements were scaled by the bond length *l_c_*, and rigidities were scaled by the fiber stretching modulus α. This meant that for a fiber of length *l_c_*, α ∼ *Er*^2^/*l_c_* and the scaled bending rigidity $\kappa ∼Er^{4}/l_{c}^{3}$; the bending-to-stretching ratio was given by $\kappa /\alpha ∼r^{2}/l_{c}^{2}∼10^{-4}$ for *l_c_* = 1 μm. (*13*). The elasticity of a densely connected network is stretching-dominated, and we expected the shear modulus to scale as $G∼\frac{\alpha }{l_{c}}∼\frac{Er^{2}}{l_{c}^{2}}∼10^{5}$ Pa, setting the order of magnitude for *c*. To inform an estimate of the stiffness for the HA gel, we used the data of Holmes *et al.* (*11*), who found the concentration of hyaluronic acid in articular cartilage to be of the order of 1 mg/ml, with a molecular mass on the order of 1 MDa. The classical estimate for a shear modulus of an aggregate of Gaussian chains, with a concentration of *n* chains per unit volume, is ∼*k_B_Tn* (*66*), yielding a shear modulus of the order of 1 kPa. These results suggested that the normalized ratio of shear modulus to stretching stiffness, μ_0_*l_c_*/α, should be ∼10^−2^. As the product of *c* and μ_0_ should yield a modulus of the order 10^3^ Pa, this separately suggests that *c* should be of order 10^5^.

We fitted for *c*, β, γ, μ_0_, and κ, using the above considerations to constrain our search. Parameters were chosen using χ^2^ minimization via the Nelder-Mead simplex algorithm in Mathematica. We found optimal values of *c* = 2.33 × 10^6^ Pa, μ_0_ = 0.001, β = 2.52 × 10^−3^ ml/mg, γ = 1.49 × 10^−4^ ml/mg, κ = 0.01, and δ = 0.0052 ml/mg, with χ^2^/*D*. *O*. *F* = 3.58. The χ^2^ value for each data bin was computed using the log base 10 of the experimental and model shear moduli. Our choice for δ was the reciprocal of the maximum observed collagen concentration, $\rho_{a,\text{max}}^{-1}$, such that *p* = 1 at this concentration. When δ was allowed to vary, we found that the reciprocal of maximum collagen concentration still proved optimal. The fact that the same values of κ, α, and δ fit both the healthy and degraded data best lends support to the idea that trypsin affects the mechanics of cartilage primarily through aggrecan degradation. See the Supplementary Materials for further details.

## Supplementary Materials

### This PDF file includes:

Supplementary Materials Figs. S1 to S3 References

## Notes

View/request a protocol for this paper from *Bio-protocol*.
