# Supplementary material for: Structural origins of cartilage shear mechanics
Source: Sci Adv. 2022 Feb 11;8(6):eabk2805. doi: 10.1126/sciadv.abk2805 (PMC8836800; doi:10.1126/sciadv.abk2805)
Supplement: Supplementary file 1 — Supplementary Materials Figs. S1 to S3 References [file sciadv.abk2805_sm.pdf]

Science Advances
AAAS

Supplementary Materials for

Structural origins of cartilage shear mechanics

Thomas Wyse Jackson*, Jonathan Michel, Pancy Lwin, Lisa A. Fortier, Moumita Das, Lawrence J. Bonassar, Itai Cohen*

*Corresponding author. Email: tsw64@cornell.edu (T.W.J.); ic64@cornell.edu (T.C.)

Published 11 February 2022, *Sci. Adv.* **8**, eabk2805 (2022)
DOI: 10.1126/sciadv.abk2805

This PDF file includes:

Supplementary Materials
Figs. S1 to S3
References

S1 Supplementary materials

S1.1 The previous model fails to capture degraded tissue mechanics

The model described previously in *Silverberg et al.* (*13*) contained a term μ for the reinforcing background modulus, representing the aggrecan matrix, however when it was compared to the experimental data for degraded tissue, we found that it was unable to describe the mechanics of the tissue when $\mu = 0$ (Fig. S1)

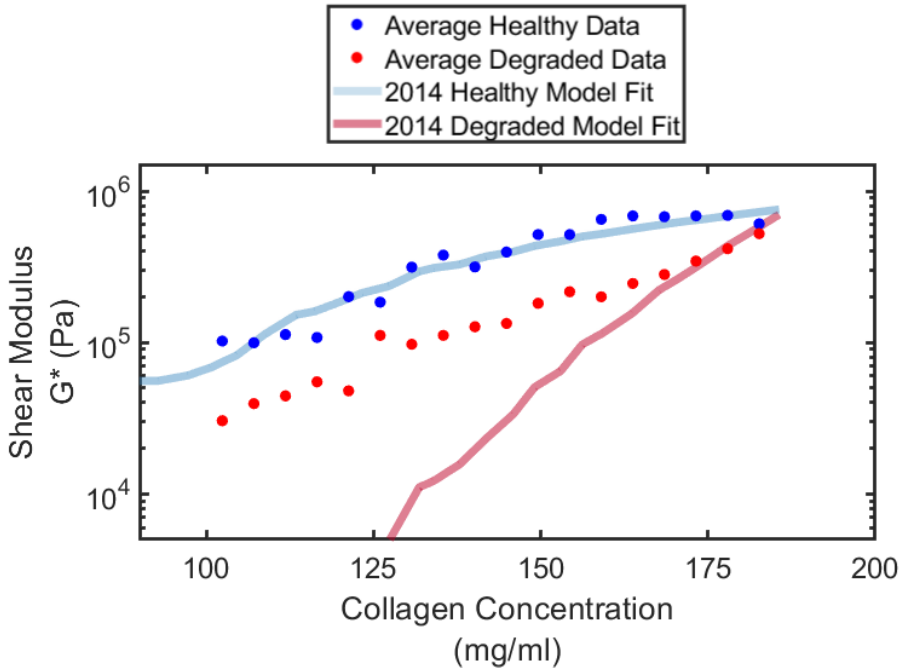

Figure S1: **Prior Models Were Not Capable of Capturing the Degraded Experimental Data.**Fits from the previous version of the model. The model was not capable of fitting the degraded curves.

S1.2 Storage and loss modulus in healthy and degraded tissue

We separated our complex modulus G* into the storage modulus (G') and loss modulus (G"). Figure S2 shows that the storage modulus is consistently larger than the loss modulus in both the healthy and degraded tissue. We find that the change in complex modulus during degradation primarily arises from a reduction in the storage modulus of the tissue.

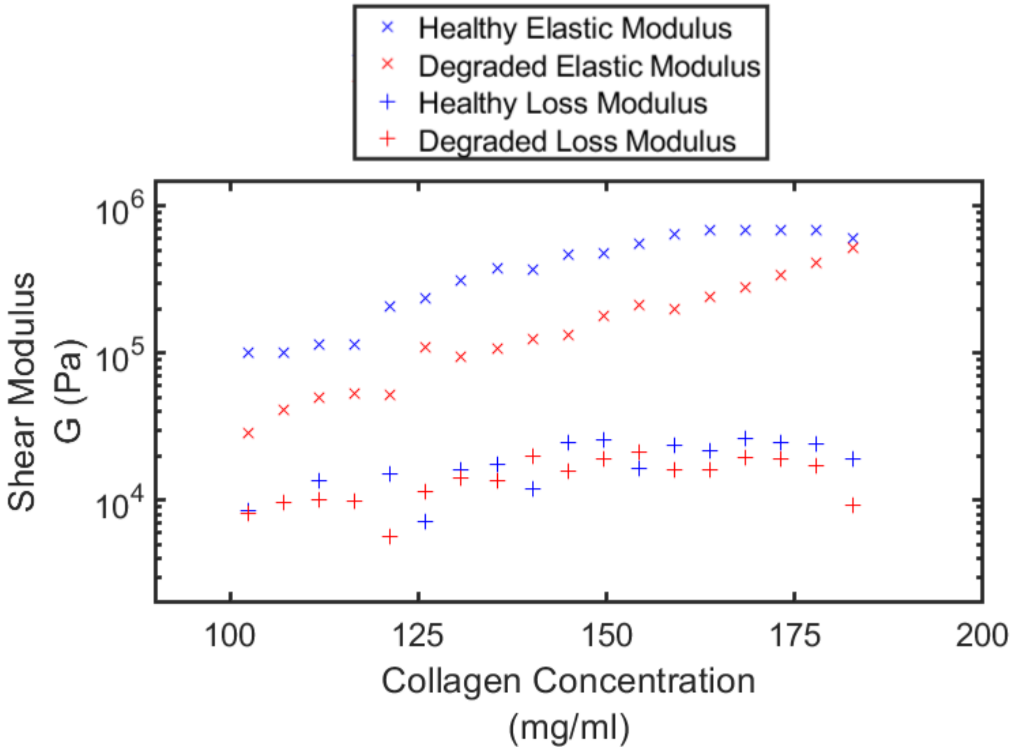

Figure S2: **The Separate Storage and Loss Moduli From the Healthy and Degraded Tissue.** The storage modulus changes drastically between the healthy and degraded tissue, whereas the loss modulus remains relatively constant.

S1.3 Simulation details for the rigidity percolation model

The model consists of a disordered kagome network made of stiff collagen fibers embedded in a continuum elastic background gel made of aggrecan and hyaluronic acid (Fig.S3), constructed following the procedure described in (*13*). The bonds in the network are randomly removed with probability $1 - p$, where $0 < p < 1$, and a continuous series of colinear bonds constitute a fiber. The fibers have a spring stiffness α and a bending modulus κ. The background gel resists fiber deformations in the transverse direction and its elasticity is described by the shear modulus μ. The energy cost of deforming this network in the linear response regime is given by:

$$
\begin{aligned}
E &= \frac{\alpha}{2} \sum_{\langle ij \rangle} p_{ij} \left(\mathbf{u}_{ij} \cdot \hat{\mathbf{r}}_{ij} \right)^2 \\
&\quad + \frac{\kappa}{2} \sum_{\langle ijk \rangle} p_{ij} p_{jk} \left[\left(\mathbf{u}_{ji} + \mathbf{u}_{jk} \right) \times \hat{\mathbf{r}}_{ji} \right]^2 \\
&\quad + \frac{\mu}{2} \sum_{\langle ij \rangle} p_{ij} \left[\mathbf{u}_{ij}^2 - \left(\mathbf{u}_{ij} \cdot \hat{\mathbf{r}}_{ij} \right)^2 \right] ,
\end{aligned} \tag{1}
$$

where the first term corresponds to the energy cost of fiber stretching, the second term to fiber bending, and the third term to deformation of the background gel (*13*).

The indices i, j, k are summed over all nodes in the network, with p_{ij} defined to be 1 when a bond between lattice sites i and j is present and 0 if such a bond is not present. The quantities $\hat{\mathbf{r}}_{ij}$ and $\mathbf{u}_{ij} = \mathbf{u}_i - \mathbf{u}_j$ are respectively the unit vector along bond ij and the corresponding relative displacement. The bond length was set to unity in Eq.(2), which renormalizes the units of distance used in calculating each elastic coefficient. Simulations were carried out for the parameter ranges $10^{-4} < \kappa/\alpha < 10^{-2}$, and $10^{-3} < \mu/\alpha < 1$. The applied compressive strain was 5%, while a range of shear strains centered about 1% were applied to compute the linear response. We adopted a simple shear protocol in which external deformations were applied along the top boundary, vertices on the bottom boundary were fixed, and periodic boundary conditions were used for the left and right sides of the network. For each set of parameters, five

networks containing $\sim 10^5$ nodes were randomly generated with a fraction $1-p$ of bonds missing, with p varied from .4 to 1 in steps of .05. The deformation energy (Eq.2) was minimized for the applied macroscopic compression and shear, and the shear modulus was calculated as a function of the bond occupation probability p (*13*).

S1.4 Energy minimization of the rigidity percolation model

To find an energy minimum, we note that the energy in (1) is quadratic in the components of the $2N$–dimensional displacement field, allowing the energy to be written as a bilinear form with a stiffness matrix, $\mathbf{K}$:

$$E = \frac{1}{2}\sum_{l=1}^{2N}\sum_{m=1}^{2N} K_{l,m}u_l u_m, \tag{2}$$

and seek a zero force state, given by

$$F = -\mathbf{K}\vec{u} + \vec{F}_C, \tag{3}$$

where $\vec{F}_C$ is a vector of constraint forces needed to impose a purely affine displacement field upon top and bottom nodes.

To isolate for nodes not on the top or bottom, here referred to as interior nodes, we define a projection operator, $P_{N\to R}$ from the $2N$–dimensional space of all displacement field components to the $2R$–dimensional space of displacements of the R interior nodes. We also define an operator $P_{R\to N}$ from the $2R$ to the $2N$–dimensional space such that, for a full $2N$–dimensional displacement vector $\vec{u}$

$$(P_{R\to N}P_{N\to R}\vec{u})_i = \begin{cases} u_i, i \text{ an interior coordinate} \\ 0, i \text{ a boundary coordinate} \end{cases} \tag{4}$$

We next separate the displacement field into two components: an affine part, $\vec{u}_A$, and a non-affine part, $\vec{u}_{NA}$, where, for a vertex with initial coordinates (x, y),

$$\vec{u}_A(x, y) = (\epsilon_s y, -\epsilon_c y), \tag{5}$$

where ϵ_s and ϵ_c are the shear and compressive strain, respectively. For well-connected networks, we expect the displacement field yielding an energetic ground state to very nearly equal

the affine displacement field, while for networks near the rigidity percolation threshold, large departures are possible (*67*).

We look for a non-affine displacement vector that leads to a zero overall force on all interior nodes. Denoting by $\vec{u}_R$ the projection of the displacement field in the reduced, $2R$–dimensional subspace, we solve

$$P_{N\to R}\mathbf{K}P_{R\to N}\vec{u}_{R,NA} = -P_{N\to R}\mathbf{K}\vec{u}_A, \tag{6}$$

using the QR factorization method in SuiteSparse (*68*). Notably, this solver can find pseudoinverse solutions even when the left-hand matrix is rank deficient, and we verified upon solution that residual forces were of order 10^{-12} or less. After obtaining a solution to equation (6), we compute the full displacement field, $\vec{u}$, as

$$\vec{u} = P_{R\to N}\vec{u}_{R,NA} + \vec{u}_A, \tag{7}$$

and obtain the strain energy according to (1).

We define the shear modulus as the second partial derivative of strain energy with respect to shear strain, ϵ_s, at fixed compressive strain $\epsilon_c = 5\%$:

$$G = \frac{1}{A}\frac{\partial^2 E}{\partial \epsilon_s^2}\bigg|_{\epsilon_c=5\%}. \tag{8}$$

The second derivative was approximated using a centered finite difference approximation, with shear strains ranging from $.8\%$ to 1.2%, in steps of $\delta\epsilon = .1\%$, according to:

$$\frac{\partial^2 E}{\partial \epsilon_s^2} \approx \frac{1}{12\delta\epsilon^2}\Bigg[16\left[u(\epsilon_s^* - \delta\epsilon) + u(\epsilon_s^* + \delta\epsilon)\right] - u(\epsilon_s^* - 2\delta\epsilon) - u(\epsilon_s^* + 2\delta\epsilon) - 30u(\epsilon_s^*)\Bigg] + \mathcal{O}(\delta\epsilon^4), \tag{9}$$

where ϵ_s^* denotes the target strain of 1%.

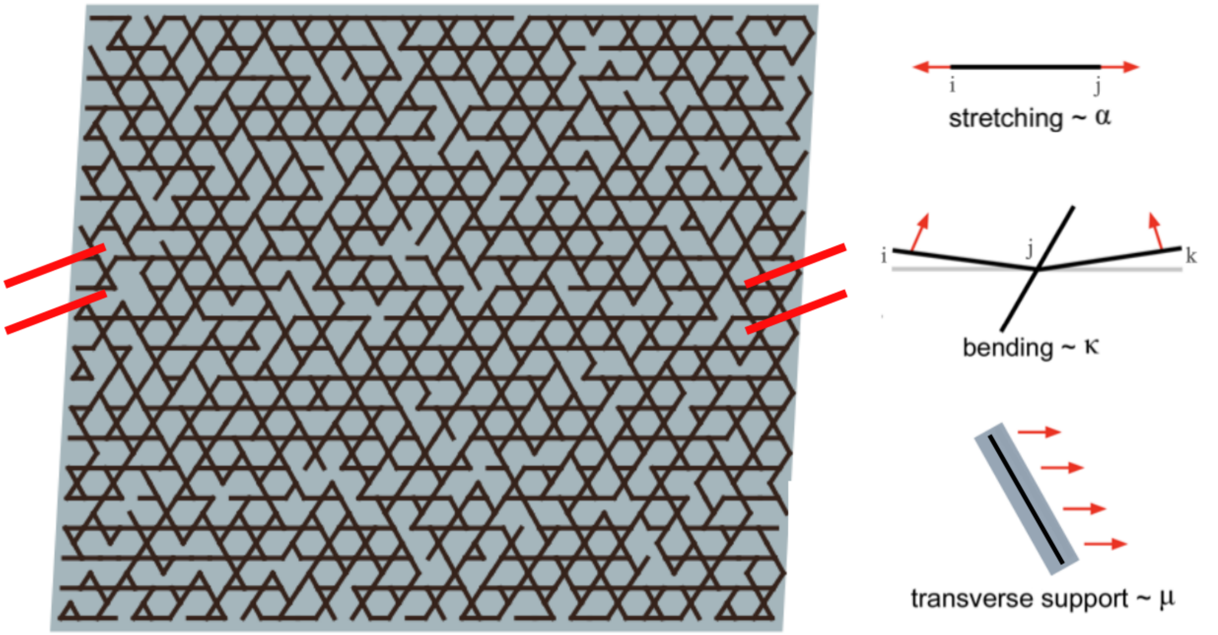

Figure S3: **Rigidity Percolation Model.** Shown is a schematic of a portion of the simulated kagome network. Hash marks indicate continuous boundary conditions in the lateral dimension. The black links represent the collagen fibers and the gray represents the background aggrecan gel. The links or bonds in the network are characterized by a stretching modulus α and a bending stiffness κ. The background gel further couples the links in the network through inhibition of transverse transport. This gel is characterized by a modulus μ.

S1.5 Fitting simulation parameters to experimental data

Experimental data were binned according to collagen concentration, with bin widths of 10 mg/ml. Within each bin, we found the arithmetic mean of collagen concentration and aggrecan concentration, and the geometric mean of shear modulus. The uncertainty in the shear modulus was estimated using the geometric standard deviation.

From discrete simulation data, we constructed a fourth-order interpolation to estimate the shear modulus of the tissue, which was taken to be a sum of a background shear modulus, μ, and a simulated shear modulus, $G_{sim}(p, \kappa, \mu)$, where κ and p denote bond bending stiffness and bond occupation probability, respectively. The shear modulus, μ, is presumed to have a base value of μ_0. We expand μ to linear order in both aggrecan concentration, ρ_a, and collagen concentration, ρ_c. We compute the full shear modulus as

$$G = c\left[(\mu_0 + \beta\rho_a + \gamma\rho_c) + G_{sim}(\delta\rho_c, \kappa, \mu_0 + \beta\rho_a + \gamma\rho_c)\right] \tag{10}$$

Here, c is a scaling factor from simulation to experimental units, β and γ are expansion coefficients capturing the reinforcement of the bare hyaluronic acid gel by proteoglycans and collagen, and δ relates collagen concentration to bond occupation probability. We model p as linearly dependent upon collagen concentration, such that the maximum experimentally observed collagen concentration corresponds with $p = 1$.

The χ^2 statistic is computed by comparing the $\log_{10}$ of the geometric mean of the shear modulus for each bin to the model value predicted using the arithmetic means of collagen and aggrecan concentrations. We optimize fitting parameters by minimizing

$$\chi^2 = \sum_{i=1}^{N} \frac{\left[\log_{10}(\bar{G}_{exp,i}) - \log_{10}(G_{model}(\bar{\rho}_{c,i}, \bar{\rho}_{a,i}))\right]^2}{\log_{10}(\sigma_i)^2}, \tag{11}$$

where N is the number of bins, $\bar{G}_{exp,i}$ is the geometric mean of the shear modulus for the ith bin, $\bar{\rho}_{c,i}$ and $\bar{\rho}_{a,i}$ are the arithmetic means of collagen and aggrecan concentrations for the ith

bin, and σ_i is the geometric standard deviation of the shear modulus in the ith bin.

We fit our model to degraded data to obtain values for c, μ_0, β γ, and κ, while setting the value of the δ to be the reciprocal of the maximum collagen concentration encountered in experiment. Parameters were constrained to be within physiologically and experimentally relevant ranges, and were optimized using χ^2 minimization via the Nelder-Mead simplex algorithm in Mathematica. We obtained the value of β by fitting the model to healthy data, while using the values obtained from degraded data for all other parameters. We found optimal values of $c = 2.33 \times 10^6$ Pa, $\mu_0 = .001$, $\beta = .0025$ ml / mg, $\gamma = 1.49 \times 10^{-4}$ ml / mg, $\kappa = .01$ and $\delta = .0052$, with $\chi^2/D.O.F = 3.58$. We tested for possible degradation of collagen by attempting to fit for a separate bending rigidity of collagen for healthy tissue, and found a negligible change.

We finally considered the possibility of treating the network and gel entirely separately, so that the third sum in (1) is eliminated, and the shear modulus is modeled according to

$$G = c \left[\mu_0 + \beta\rho_a + \gamma\rho_c + G_{sim} \left(\delta\rho_c, \kappa \right) \right]. \tag{12}$$

We optimized parameters as described above, finding $c = 2.37 \times 10^6 Pa$, $\mu_0 = 10^{-3}$, $\beta = 5.07 \times 10^{-4}$ ml / mg, $\gamma = 3.58 \times 10^{-4} ml/mg$, $\kappa = 10^{-2}$, and $\delta = 5.2 \times 10^{-3}$, with $\chi^2/D.O.F. = 6.7$. This model exhibited considerably worse agreement with experimental observations than the approach that considers direct coupling between the network and background gel. In particular, ignoring direct coupling leads to predictions for the shear modulus that are systematically too high for low collagen concentration. This suggests that ignoring the interaction between the collagen scaffold and the aggrecan matrix eliminates an important means of reinforcing cartilage, leading to an overprediction of the shear modulus of aggrecan to compensate for this deficit. We thus fund the coupling term in the third sum of (1) to significantly improve the predictive power of our model.

S1.6 Assessment of model prediction based on prior work

We can also assess the reasonableness of our estimate for β via comparison with previously published results. When our model is evaluated with the above fitting parameters (i.e. aggrecan concentration of $\sim$36.7 ml/mg and no collagen), we obtain a shear modulus of 218 kPa. Noteably, Jin and Grodzinsky evaluated the electrostatic contribution to the shear modulus of cartilage and found a similar contribution ($\sim$250 kPa) (*23*).

REFERENCES AND NOTES

1. J. C. Mora, R. Przkora, Y. Cruz-Almeida, Knee osteoarthritis: Pathophysiology and current treatment modalities. *J. Pain Res.* **11**, 2189–2196 (2018).

2. E. Ayhan, H. Kesmezacar, I. Akgun, Intraarticular injections (corticosteroid, hyaluronic acid, platelet rich plasma) for the knee osteoarthritis. *World J. Orthop.* **5**, 351–361 (2014).

3. Y. Krishnan, A. J. Grodzinsky, Cartilage diseases. *Matrix Biol.* **71-72**, 51–69 (2018).

4. J. Shen, Y. Abu-Amer, R. J. O'Keefe, A. McAlinden, Inflammation and epigenetic regulation in osteoarthritis. *Connect. Tissue Res.* **58**, 49–63 (2017).

5. F. Berenbaum, Osteoarthritis as an inflammatory disease (osteoarthritis is not osteoarthrosis!). *Osteoarthr. Cartil.* **21**, 16–21 (2013).

6. G. E. Kempson, M. A. Freeman, S. A. Swanson, Tensile properties of articular cartilage. *Nature* **220**, 1127–1128 (1968).

7. A. K. Williamson, A. C. Chen, K. Masuda, E. J.-M. A. Thonar, R. L. Sah, Tensile mechanical properties of bovine articular cartilage: Variations with growth and relationships to collagen network components. *J. Orthop. Res.* **21**, 872–880 (2003).

8. J. P. Wu, T. B. Kirk, M. H. Zheng, Study of the collagen structure in the superficial zone and physiological state of articular cartilage using a 3D confocal imaging technique. *J. Orthop. Surg. Res.* **3**, 29 (2008).

9. J. Oinas, A. P. Ronkainen, L. Rieppo, M. A. J. Finnilä, J. T. Iivarinen, P. R. van Weeren, H. J. Helminen, P. A. J. Brama, R. K. Korhonen, S. Saarakkala Composition, structure and tensile biomechanical properties of equine articular cartilage during growth and maturation. *Sci. Rep.* **8**, 11357 (2018).

10. R. Boyanich, T. Becker, F. Chen, T. B. Kirk, G. Allison, J.-P. Wu Application of confocal, SHG and atomic force microscopy for characterizing the structure of the most superficial layer of articular cartilage. *J. Microsc.* **275**, 159–171 (2019).

11. M. W. A. Holmes, M. T. Bayliss, H. Muir, Hyaluronic acid in human articular cartilage. Age-related changes in content and size. *Biochem. J.* **250**, 435–441 (1988).

12. G. E. Kempson, H. Muir, C. Pollard, M. Tuke, The tensile properties of the cartilage of human femoral condyles related to the content of collagen and glycosaminoglycans. *Biochim. Biophys. Acta Gen. Subj.* **297**, 456–472 (1973).

13. J. Silverberg, A. R. Barrett, M. Das, P. B. Petersen, L. J. Bonassar, I. Cohen, Structure-function relations and rigidity percolation in the shear properties of articular cartilage. *Biophys. J.* **107**, 1721–1730 (2014).

14. L. J. Bonassar, E. H. Frank, J. C. Murray, C. G. Paguio, V. L. Moore, M. W. Lark, J. D. Sandy, J. J. Wu, D. R. Eyre, A. J. Grodzinsky Changes in cartilage composition and physical properties due to stromelysin degradation. *Arthritis Rheum.* **38**, 173–183 (1995).

15. A. W. Palmer, C. G. Wilson, E. J. Baum, M. E. Levenston, Composition-function relationships during IL-1-induced cartilage degradation and recovery. *Osteoarthr. Cartil.* **17**, 1029–1039 (2009).

16. C. G. Wilson, A. W. Palmer, F. Zuo, E. Eugui, S. Wilson, R. Mackenzie, J. D. Sandy, M. E. Levenston Selective and non-selective metalloproteinase inhibitors reduce IL-1-induced cartilage degradation and loss of mechanical properties. *Matrix Biol.* **26**, 259–268 (2007).

17. L. A. Setton, D. M. Elliott, V. C. Mow, Altered mechanics of cartilage with osteoarthritis: Human osteoarthritis and an experimental model of joint degeneration. *Osteoarthr. Cartil.* **7**, 2–14 (1999).

18. E. H. Frank, A. J. Grodzinsky, Cartilage electromechanics—II. A continuum model of cartilage electrokinetics and correlation with experiments. *J. Biomech.* **20**, 629–639 (1987).

19. V. C. Mow, S. C. Kuei, W. M. Lai, C. G. Armstrong, Biphasic creep and stress relaxation of articular cartilage in compression: Theory and experiments. *J. Biomech. Eng.* **102**, 73–84 (1980).

20. M. D. Buschmann, A. J. Grodzinsky, A molecular model of proteoglycan-associated electrostatic forces in cartilage mechanics. *J. Biomed. Eng.* **117**, 179–192 (1995).

21. P. J. Basser, R. Schneiderman, R. A. Bank, E. Wachtel, A. Maroudas, Mechanical properties of the collagen network in human articular cartilage as measured by osmotic stress technique. *Arch. Biochem. Biophys.* **351**, 207–219 (1998).

22. J. Rieppo, J. Töyräs, M. T. Nieminen, V. Kovanen, M. M. Hyttinen, R. K. Korhonen, J. S. Jurvelin, H. J. Helminen Structure-function relationships in enzymatically modified articular cartilage. *Cells Tissues Organs* **175**, 121–132 (2003).

23. M. Jin, A. J. Grodzinsky, Effect of electrostatic interactions between glycosaminoglycans on the shear stiffness of cartilage: A molecular model and experiments. *Macromolecules* **34**, 8330–8339 (2001).

24. C. R. Henak, G. A. Ateshian, J. A. Weiss, Finite element prediction of transchondral stress and strain in the human hip. *J. Biomech. Eng.* **136**, 021021 (2014).

25. T. F. Besier, G. E. Gold, S. L. Delp, M. Fredericson, G. S. Beaupré, The influence of femoral internal and external rotation on cartilage stresses within the patellofemoral joint. *J. Orthop. Res.* **26**, 1627–1635 (2008).

26. D. A. Head, F. C. MacKintosh, A. J. Levine, Nonuniversality of elastic exponents in random bond-bending networks. *Phys. Rev. E* **68**, 025101 (2003).

27. B. J. Gurmessa, N. Bitten, D. T. Nguyen, O. A. Saleh, J. L. Ross, M. Das, R. M. Robertson-Anderson, Triggered disassembly and reassembly of actin networks induces rigidity phase transitions. *Soft Matter* **15**, 1335–1344 (2019).

28. D. J. Griffin, J. Vicari, M. R. Buckley, J. L. Silverberg, I. Cohen, L. J. Bonassar Effects of enzymatic treatments on the depth-dependent viscoelastic shear properties of articular cartilage. *J. Orthop. Res.* **32**, 1652–1657 (2014).

29. J. M. Middendorf, C. Dugopolski, S. Kennedy, E. Blahut, I. Cohen, L. J. Bonassar Heterogeneous matrix deposition in human tissue engineered cartilage changes the local shear modulus and resistance to local construct buckling. *J. Biomech.* **105**, 109760 (2020).

30. C. D. DiDomenico, A. Kaghazchi, L. J. Bonassar, Measurement of local diffusion and composition in degraded articular cartilage reveals the unique role of surface structure in controlling macromolecular transport. *J. Biomech.* **82**, 38–45 (2019).

31. M. S. Laasanen, J. Töyräs, J. Hirvonen, S. Saarakkala, R. K. Korhonen, M. T. Nieminen, I. Kiviranta, J. S. Jurvelin Novel mechano-acoustic technique and instrument for diagnosis of cartilage degeneration. *Physiol. Meas.* **23**, 491–503 (2002).

32. P. A. Hardy, A. C. Ridler, C. B. Chiarot, D. B. Plewes, R. M. Henkelman, Imaging articular cartilage under compression–cartilage elastography. *Magn. Reson. Med.* **53**, 1065–1073 (2005).

33. A. Kotelsky, C. W. Woo, L. F. Delgadillo, M. S. Richards, M. R. Buckley, An alternative method to characterize the quasi-static, nonlinear material properties of murine articular cartilage. *J. Biomech. Eng.* **140**, 0110071–0110079 (2017).

34. Z.-q. Liu, F. Y. Tuo, L. Song, Y. X. Liu, X. P. Dong, D. M. Li, D. Y. Zhou, F. Shahidi Action of trypsin on structural changes of collagen fibres from sea cucumber (*Stichopus japonicus*). *Food Chem.* **256**, 113–118 (2018).

35. A. Chang, S. Y. Tang, Determination of the depth- and time- dependent mechanical behavior of mouse articular cartilage using cyclic reference point indentation. *Cartilage* **11**, 358–363 (2020).

36. T. Hafner, J. Schock, M. Post, D. B. Abrar, P. Sewerin, K. Linka, M. Knobe, C. Kuhl, D. Truhn, S. Nebelung A serial multiparametric quantitative magnetic resonance imaging study to assess proteoglycan depletion of human articular cartilage and its effects on functionality. *Sci. Rep.* **10**, 15106 (2020).

37. V. Virtanen, E. Nippolainen, R. Shaikh, I. O. Afara, J. Töyräs, J. Solheim, V. Tafintseva, B. Zimmermann, A. Kohler, S. Saarakkala, L. Rieppo, Infrared fiber-optic spectroscopy detects bovine articular cartilage degeneration. *Cartilage* **13**, 285S–294S (2021).

38. M. R. Buckley, J. P. Gleghorn, L. J. Bonassar, I. Cohen, Mapping the depth dependence of shear properties in articular cartilage. *J. Biomech.* **41**, 2430–2437 (2008).

39. M. R. Buckley, A. J. Bergou, J. Fouchard, L. J. Bonassar, I. Cohen, High-resolution spatial mapping of shear properties in cartilage. *J. Biomech.* **43**, 796–800 (2010).

40. M. R. Buckley, L. J. Bonassar, I. Cohen, Localization of viscous behavior and shear energy dissipation in articular cartilage under dynamic shear loading. *J. Biomech. Eng.* **135**, 31002 (2013).

41. J. L. Silverberg, S. Dillavou, L. Bonassar, I. Cohen, Anatomic variation of depth-dependent mechanical properties in neonatal bovine articular cartilage. *J. Orthop. Res.* **31**, 686–691 (2013).

42. A. R. Poole, I. Pidoux, A. Reiner, L. Rosenberg, An immunoelectron microscope study of the organization of proteoglycan monomer, link protein, and collagen in the matrix of articular cartilage. *J. Cell Biol.* **93**, 921–937 (1982).

43. V. K. Yadavalli, D. V. Svintradze, R. M. Pidaparti, Nanoscale measurements of the assembly of collagen to fibrils. *Int. J. Biol. Macromol.* **46**, 458–464 (2010).

44. V. C. Mow, A. Ratcliffe, A. Robin Poole, Cartilage and diarthrodial joints as paradigms for hierarchical materials and structures. *Biomaterials* **13**, 67–97 (1992).

45. X. Mao, O. Stenull, T. C. Lubensky, Elasticity of a filamentous kagome lattice. *Phys. Rev. E* **87**, 042602 (2013).

46. D. Burstein, A. Bashir, M. L. Gray, MRI techniques in early stages of cartilage disease. *Invest. Radiol.* **35**, 622–638 (2000).

47. D. J. Griffin, E. D. Bonnevie, D. J. Lachowsky, J. C. A. Hart, H. D. Sparks, N. Moran, G. Matthews, A. J. Nixon, I. Cohen, L. J. Bonassar Mechanical characterization of matrix-induced autologous chondrocyte implantation (MACI®) grafts in an equine model at 53 weeks. *J. Biomech.* **48**, 1944–1949 (2015).

48. C. R. Henak, K. A. Ross, E. D. Bonnevie, L. A. Fortier, I. Cohen, J. G. Kennedy, L. J. Bonassar Human talar and femoral cartilage have distinct mechanical properties near the articular surface. *J. Biomech.* **49**, 3320–3327 (2016).

49. M. Brittberg, A. Lindahl, A. Nilsson, C. Ohlsson, O. Isaksson, L. Peterson Treatment of deep cartilage defects in the knee with autologous chondrocyte transplantation. *N. Engl. J. Med.* **331**, 889–895 (1994).

50. A. S. G. van Oosten, X. Chen, L. K. Chin, K. Cruz, A. E. Patteson, K. Pogoda, V. B. Shenoy, P. A. Janmey Emergence of tissue-like mechanics from fibrous networks confined by close-packed cells. *Nature* **573**, 96–101 (2019).

51. R. Roy, A. Boskey, L. J. Bonassar, Processing of type I collagen gels using nonenzymatic glycation. *J. Biomed. Mater. Res. A* **93**, 843–851 (2010).

52. L. Slyker, N. Diamantides, J. Kim, L. J. Bonassar, Mechanical performance of collagen gels is dependent on purity, α1/α2 ratio, and telopeptides. *J. Biomed. Mater. Res. A* **110**, 11–20 (2022).

53. D. K. Temple, A. A. Cederlund, B. M. Lawless, R. M. Aspden, D. M. Espino, Viscoelastic properties of human and bovine articular cartilage: A comparison of frequency-dependent trends. *BMC Musculoskelet. Disord.* **17**, 419 (2016).

54. K. Potter, L. H. Kidder, I. W. Levin, E. N. Lewis, R. G. S. Spencer, Imaging of collagen and proteoglycan in cartilage sections using Fourier transform infrared spectral imaging. *Arthritis Rheum.* **44**, 846–855 (2001).

55. N. P. Camacho, P. West, P. A. Torzilli, R. Mendelsohn, FTIR microscopic imaging of collagen and proteoglycan in bovine cartilage. *Biopolymers* **62**, 1–8 (2001).

56. B. C. Smith, *Fundamentals of Fourier Transform Infrared Spectroscopy* (CRC Press, 1995).

57. Y. Luo, D. Sinkeviciute, Y. He, M. Karsdal, Y. Henrotin, A. Mobasheri, P. Önnerfjord, A. Bay-Jensen The minor collagens in articular cartilage. *Protein Cell* **8**, 560–572 (2017).

58. C. D. Hoemann, Molecular and Biochemical Assays of Cartilage Components, in *Cartilage and Osteoarthritis: Volume 2: Structure and In Vivo Analysis*, F. De Ceuninck, M. Sabatini, P. Pastoureau, Eds. (Methods in Molecular Medicine, Humana Press, 2004), pp. 127–156.

59. L. Zhang, D. Z. Rocklin, B. G. G. Chen, X. Mao, Rigidity percolation by next-nearest-neighbor bonds on generic and regular isostatic lattices. *Phys. Rev. E* **91**, 032124 (2015).

60. D. B. Liarte, O. Stenull, X. Mao, T. C. Lubensky, Elasticity of randomly diluted honeycomb and diamond lattices with bending forces. *J. Phys. Condens. Matter* **28**, 165402 (2016).

61. D. B. Liarte, X. Mao, O. Stenull, T. C. Lubensky, Jamming as a multicritical point. *Phys. Rev. Lett.* **122**, 128006 (2019).

62. C. P. Broedersz, X. Mao, T. C. Lubensky, F. C. MacKintosh, Criticality and isostaticity in fibre networks. *Nat. Phys.* **7**, 983–988 (2011).

63. M. Das, F. C. MacKintosh, A. J. Levine, Effective medium theory of semiflexible filamentous networks. *Phys. Rev. Lett.* **99**, 038101 (2007).

64. E. Berthier, J. E. Kollmer, S. E. Henkes, K. Liu, J. M. Schwarz, K. E. Daniels Rigidity percolation control of the brittle-ductile transition in disordered networks. *Phys. Rev. Mater.* **3**, 075602 (2019).

65. A. K. Jeffery, G. W. Blunn, C. W. Archer, G. Bentley, Three-dimensional collagen architecture in bovine articular cartilage. *J. Bone Joint Surg. Br.* **73**, 795–801 (1991).

66. R. A. Pethrick, Polymer physics. Edited by Michael Rubinstein and Ralph H Colby Oxford University Press, 2003. ISBN 019852059X. pp 440. *Polym. Int.* **53**, 1394–1395 (2004).

67. B. A. DiDonna, T. C. Lubensky, Nonaffine correlations in random elastic media. *Phys. Rev. E* **72**, 066619 (2005).

68. T. A. Davis, Algorithm 915, SuiteSparseQR: Multifrontal multithreaded rank-revealing sparse QR factorization. *ACM Trans. Math. Softw.* **38**, 1–22 (2011).
